# Primary Staging of Prostate Cancer Patients with [^18^F]PSMA-1007 PET/CT Compared with [^68^Ga]Ga-PSMA-11 PET/CT

**DOI:** 10.3390/jcm11175064

**Published:** 2022-08-29

**Authors:** Manuela A. Hoffmann, Jonas Müller-Hübenthal, Florian Rosar, Nicolas Fischer, Finn Edler von Eyben, Hans-Georg Buchholz, Helmut J. Wieler, Mathias Schreckenberger

**Affiliations:** 1 Department of Occupational Health & Safety, Federal Ministry of Defense, 53123 Bonn, Germany; 2 Clinic of Nuclear Medicine, Johannes Gutenberg-University, 55101 Mainz, Germany; 3 Practice of Radiology and Nuclear Medicine, Praxis im KölnTriangle, 50679 Cologne, Germany; 4 Department of Nuclear Medicine, Saarland University Medical Center, 66421 Homburg, Germany; 5 Clinic of Urology, Medical Center of Leverkusen, 51375 Leverkusen, Germany; 6 Center for Tobacco Control Research, DK-5230 Odense, Denmark; 7 Medical Center, University of Dusseldorf, 40225 Dusseldorf, Germany

**Keywords:** PSMA hybrid imaging, staging of primary prostate cancer, [^18^F]PSMA-1007, [^68^Ga]Ga-PSMA-11, SUV_max_ cut-off level, prostate carcinoma

## Abstract

Background: Hybrid imaging with prostate-specific membrane antigen (PSMA) is gaining importance as an increasingly meaningful tool for prostate cancer (PC) diagnostics and as a guide for therapy decisions. This study aims to investigate and compare the performance of [^18^F]PSMA-1007 (^18^F-PSMA) and [^68^Ga]Ga-PSMA-11 positron emission tomography/computed tomography (^68^Ga-PSMA) in the initial staging of PC patients. Methods: The data of 88 biopsy-proven patients were retrospectively evaluated. PSMA-avid lesions were compared with the histopathologic Gleason Score (GS) for prostate biopsies, and the results were plotted by receiver operating characteristic (ROC)-curve. Optimal maximum standardized uptake value (SUV_max_) cut-off values were rated using the Youden index. Results: ^18^F-PSMA was able to distinguish GS ≤ 7a from ≥7b with a sensitivity of 62%, specificity of 85%, positive predictive value (PPV) of 92%, and accuracy of 67% for a SUV_max_ of 8.95, whereas sensitivity was 54%, specificity 91%, PPV 93%, and accuracy 66% for ^68^Ga-PSMA (SUV_max_ 8.7). Conclusions: Both methods demonstrated a high concordance of detected PSMA-avid lesions with histopathologically proven PC. ^18^F-PSMA and ^68^Ga-PSMA are both suitable for the characterization of primary PC with a comparable correlation of PSMA-avid lesions with GS. Neither method showed a superior advantage. Our calculated SUV_max_ thresholds may represent valuable parameters in clinical use to distinguish clinically significant PC (csPC) from non-csPC.

## 1. Introduction

Prostate carcinoma (PC) is the second most common tumor in men worldwide. Its predicted mortality rate in the European Union for 2020 is 10/100,000, which has decreased by 7.1% since 2015 due to advances in screening and treatment of the disease [1]. In particular, the early detection of PC and the early initiation of therapy have contributed significantly to the reduced mortality rate. 

Current conventional imaging for PC, such as multiparametric magnetic resonance imaging (MRI) and computed tomography (CT), show limitations, especially in the primary diagnosis of lymph node metastases (LNM) [2]. Other diagnostic methods, such as positron emission tomography (PET), usually in combination with CT, are therefore used in PC diagnostics. The prospective, randomized multicenter study called “proPSMA” showed that in patients with biopsy-proven high-risk PC, PET/CT with prostate-specific membrane antigen (PSMA PET/CT) imaging is superior to conventional combined CT and bone scintigraphy for primary staging of PC metastases [2,3]. The transmembrane protein PSMA is particularly overexpressed in higher-grade prostate cancer cells and offers an optimal target for radiolabeled ligands [4]. One of the world’s most commonly used PSMA inhibitors is the ^68^Gallium(^68^Ga)-labeled [^68^Ga]Ga-HBED-CC-PSMA, also named [^68^Ga]Ga-PSMA-11, which was also used in the Hofmann study [2,3,5]. Several other PSMA ligands for labeling with ^68^Ga and ^18^Fluorine (^18^F) have been developed in recent years. In particular, the ^18^F-labeled tracers will be further explored [2,4]. ^18^F has a half-life of 110 min, whereas ^68^Ga has one of 68 min, which is an advantage for the delivery of radiopharmaceuticals. An additional advantage of ^18^F-labeled PSMA ligands is optimal positron energy, which enables higher resolution of PET images with refined image quality [2,4,6]. Currently, according to the European Association of Urology (EAU), European Association of Nuclear Medicine (EANM), European Society for Radiotherapy & Oncology (ESTRO), European Society of Urogenital Radiology (ESUR), the International Society of Urological Pathology (ISUP), and the International Society of Geriatric Oncology (SIOG) there are few comparative data on ^18^F- with ^68^Ga-labeled PSMA tracers in a clinical setting [2].

The goal of this study is to investigate and compare [^18^F]PSMA-1007 PET/CT (^18^F-PSMA) and [^68^Ga]Ga-PSMA-11 PET/CT (^68^Ga-PSMA) for the primary staging of PC patients and to distinguish between low- and intermediate-risk versus (vs.) high-risk PC as well as between low- and intermediate-favorable risk vs. intermediate-unfavorable and high-risk PC, using the best maximum standardized uptake value (SUV_max_) cut-off value to identify clinically significant PC foci.

## 2. Materials and Methods

### 2.1. Study Design

Our investigation included 88 consecutive patients with elevated serum PSA levels and with biopsy-confirmed PC who underwent PSMA PET/CT for primary staging and specifically for the detection of possible metastases. For the retrospective analysis of the data, the datasets of patients who had received prior prostate therapy were excluded. The data for the period 2017 to 2021 were collected at a practice for Radiology and Nuclear Medicine in Cologne, Germany. Fifty-two patients underwent ^18^F-PSMA, and thirty-six patients underwent ^68^Ga-PSMA. The PSMA uptake of the ^18^F-PSMA and of the ^68^Ga-PSMA PET findings were quantified as SUV_max_. The PSMA-positive lesions in the included patients were compared with histopathologic results of the prostate biopsies. A prostate biopsy was performed in all patients. PC was verified histologically with TRUS-guided or multiparametric MRI (mpMRI)-fusion guided prostate biopsy. In all patients, an adenocarcinoma of the prostate was histopathologically proven by biopsy. The biopsy results expressed as Gleason Score (GS) formed the reference basis for the PSMA PET/CT findings. Clinically significant PC (csPC) was defined as GS 7b-tumors or greater (any ISUP grade group ≥ 3) (subgroup: csPCa) and as GS 8-tumors or greater (any ISUP grade group ≥ 4) (subgroup: csPCb) [2]. 

### 2.2. Positron Emission Tomography/Computed Tomography Imaging Protocol and Interpretation

The study was performed using a PET/CT scanner (Gemini TF16; Philips Medical Systems, Best, The Netherlands). PET/CT images were acquired in 3D acquisition mode (matrix 168 × 168) 90 ± 10 min after intravenous injection of 326 ± 51.8 MBq [^18^F]PSMA-1007 or 60 ± 10 min post injectionem (p.i.) of 257 ± 85.7 MBq [^68^Ga]Ga-PSMA-11. PET images from the skull base to the proximal thigh were acquired for 3 min per bed position (axial field of view: 21.8 cm). A maximum inspiratory contrast-enhanced CT in the venous phase was performed in all included patients for attenuation correction and anatomical correlation. Decay, random, scatter, and attenuation correction were implemented. PET image reconstruction was carried out by using an ordered-subset expectation maximization (OSEM)-algorithm with 2 iterations and 14 subsets and Gaussian filtering with 4.2 mm transaxial resolution at full width at half maximum. Volumes of interest (VOIs) were drawn on the foci suspected of being malignant due to the PSMA distribution pattern on PET in consensus with CT imaging. Values for tracer uptake expressed as the SUV_max_ measured on the VOIs were plotted on a receiver operating characteristic (ROC) curve. The area under the ROC (AUC) as well as the best cut-off level for SUV_max_ to classify the VOIs were calculated. Two experienced board-certified nuclear medicine physicians and two experienced board-certified radiology physicians, each of them with more than 5 years of experience in PSMA PET/CT hybrid imaging, assessed the images by consensus. 

### 2.3. Statistical Analysis

Numeric data are presented as median or mean ± standard deviation (SD). We evaluated the relationship between PSMA PET/CT positivity (e.g., expressed as SUV_max_) and clinical parameters such as GS. To compare the two patient cohorts ^18^F-PSMA and ^68^Ga-PSMA and identify differences between them, we performed Student’s *t*-tests for data that showed a normal distribution or nonparametric Mann–Whitney U tests for sample data that was not normally distributed. Using a ROC curve analyses, the performances of the procedures (^18^F-PSMA and ^68^Ga-PSMA) for distinguishing between PC with low- and intermediate-favorable risk vs. intermediate-unfavorable and high-risk as well as between low- and intermediate-risk vs. high-risk were calculated by plotting sensitivity against 1-specificity. Optimal SUV_max_ cut-off values were rated using the Youden index for the separate methods (^18^F-PSMA and ^68^Ga-PSMA). A *p* value < 0.05 was considered as statistically significant. We carried out the statistical analyses using SPSS version 27.0 (IBM SPSS Statistics Corporation, Ehningen, Germany).

## 3. Results

We identified 88 patients who underwent ^18^F-PSMA (52) or ^68^Ga-PSMA (36). The median age was 67.5 years (range 51–80 years) in the patient group of ^18^F-PSMA and 65.5 years (range 48–79 years) in patients whose imaging was conducted with ^68^Ga-PSMA. Clinical and pathological characteristics of the study population are summarized in Table 1.

PSMA-avid lesions were found in all 52 study patients in the ^18^F-PSMA cohort and in 97.2% (35/36) of the ^68^Ga-PSMA cohort. The ^18^F-PSMA scans detected prostatic lesions with elevated PSMA avidity in 100% (52/52), LNM in 32.7% (17/52), and bone metastases in 17.3% (9/52) of cases. A total of 35 out of 36 (97.2%) untreated patients, who underwent a ^68^Ga-PSMA, showed lesions with an elevated tracer uptake in the prostate. ^68^Ga-PSMA scans also detected LNM in 16.7% (6/36) and bone metastases in 8.4% (3/36) of cases. A total of 33 patients, who underwent ^18^F-PSMA, demonstrated solitary PSMA tracer-positive prostatic lesions, whereas 26 patients showed them in the ^68^Ga-PSMA group.

**Figure 1:**
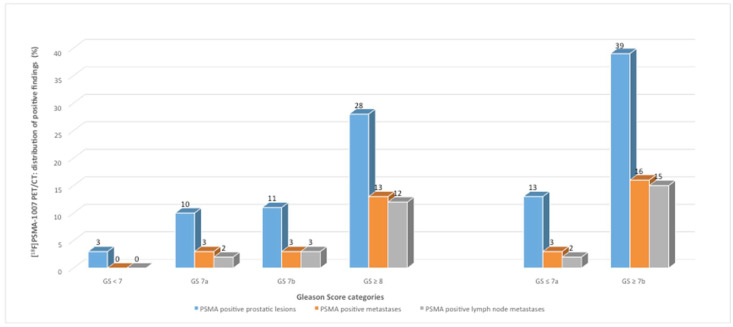
Distribution of positive findings (shown by [^18^F]PSMA-1007 PET/CT) classified by Gleason Score (GS) categories (patients with GS < 7 to GS ≥ 8 and the comparison of GS ≤ 7a versus patients with GS ≥ 7b).

**Figure 2:**
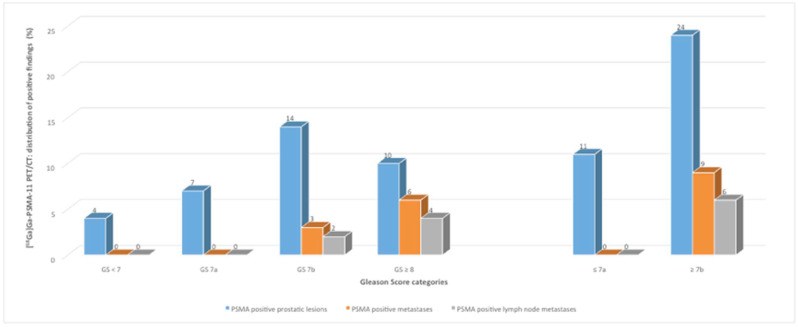
Distribution of positive findings (shown by [^68^Ga]Ga-PSMA-11 PET/CT) classified by Gleason Score (GS) categories (patients with GS < 7 to GS ≥ 8 and the comparison of GS ≤ 7a versus patients with GS ≥ 7b).

In our study, 5.8% (3/52) of PSMA-positive PET lesions, based on all patients with PSMA-positive findings, in the ^18^F-PSMA cohort and 11.4% (4/35) in the ^68^Ga-PSMA cohort were categorized as low-risk PC (GS < 7) with ISUP grade group 1. In one patient with a biopsy finding of GS 6, no increased PSMA avidity was detected in the PET/CT with [^68^Ga]Ga-PSMA-11. Intermediate-risk PC (GS 7) with ISUP grade groups 2 and 3 occurred in 40.4% (21/52) of ^18^F-PSMA-positive and in 60% (21/35) of ^68^Ga-PSMA-positive patients, whereas 53.8% (28/52, ^18^F-PSMA) and 28.6% (10/35, ^68^Ga-PSMA) showed high-risk PC lesions with an ISUP grade group 4 to 5 (Table 2, Figure 1 and Figure 2).

The 88 study patients were separately (^18^F-PSMA and ^68^Ga-PSMA) grouped into categories by GS and compared as follows: patients with GS 6 and GS 7 vs. patients with GS ≥ 8 and with GS 6 and GS 7a vs. patients with GS ≥ 7b (Figure 1 and Figure 2). 

In the ^18^F-PSMA cohort, PC prostatic lesions with histopathology of low- and intermediate-favorable risk PC (GS ≤ 7a) were shown in 25% (13/52) compared to 75% (39/52) with histopathology of intermediate-unfavorable and high-risk PC (GS ≥ 7b) (Figure 1). PSMA-avid metastases and PSMA-positive LNM were shown in 5.8% with GS ≤ 7a (3/52) vs. 30.8% with GS ≥ 7b (16/52) and in 3.8% with GS ≤ 7a (2/52) vs. 28.8% (15/52) with GS ≥ 7b (Figure 1). 

For the ^68^Ga-PSMA cohort, the distribution of the PSMA-avid PC lesions in the prostate was as follows: 31.4% (11/35) with GS ≤ 7a vs. 68.6% (24/35) with GS ≥ 7b, respectively (Figure 2). Neither PSMA-positive metastases nor LNM were shown in the subgroup with GS ≤ 7a, whereas the subgroup with GS ≥ 7b revealed PSMA-avid metastases in 25.7% (9/35) of cases and positive LNM in 17.1% (6/35) (Figure 2).

The PSMA uptake of the [^18^F]PSMA-1007 and of the [^68^Ga]Ga-PSMA-11 PET findings was quantified as SUV_max_. Comparing ^18^F-PSMA and ^68^Ga-PSMA scanned patients, there was no statistical significance for the differentiation of mean and median SUV_max_ for the most intense prostatic lesions (*p* = 0.224) (mean SUV_max_ ± SD: 12.2 ± 10.4 vs. 10.0 ± 8.0, median SUV_max_ 9.0 vs. 6.7).

When using a SUV_max_ of 2.5 as the cut-off value between PC lesions in the prostate with low- and intermediate-favorable risk (GS ≤ 7a) vs. with intermediate-unfavorable and high-risk (GS ≥ 7b), ^18^F-PSMA indicated a sensitivity of 100%, a positive predictive value (PPV) of 76%, and an accuracy of 76% (Table 3). For ^68^Ga-PSMA, the sensitivity was 97%, the PPV was 75%, and the accuracy was 77%, respectively (Table 3). 

Using the Youden index, the best analyzed cut-off value for ^18^F-PSMA was a SUV_max_ of 8.95 (subgroup: ^18^F-7a/b) for distinguishing GS ≤ 7a from GS ≥ 7b prostatic lesions. ROC analysis showed an AUC of 0.750 (95% Cl 0.590; 0.911; SD (AUC) = 0.082; *p* = 0.007) for the comparison with a SUV_max_ of 8.95 (^18^F-7a/b). The sensitivity, the specificity, the PPV, and the accuracy for ^18^F-7a/b was 62%, 85%, 92%, and 67%, respectively. For the differentiation of GS ≤ 7 from GS ≥ 8 (subgroup: ^18^F-7/8) an AUC of 0.592 (95% Cl 0.539; 0.881; SD (AUC) = 0.055; *p* = 0.26) with a SUV_max_ of 4.75 (^18^F-7/8) was evaluated with a sensitivity of 90%, a specificity of 52%, a PPV of 61%, and an accuracy of 63%, respectively (Table 3). 

By means of ROC analysis, the best cut-off value for ^68^Ga-PSMA was a SUV_max_ of 8.7 (subgroup: ^68^Ga-7a/b) to differentiate GS ≤ 7a and GS ≥ 7b PC lesions (AUC = 0.814; 95% Cl 0.668; 0.961; SD (AUC) = 0.075; *p* = 0.003) with a sensitivity of 54%, a specificity of 91%, a PPV of 93%, and an accuracy of 66%. The best AUC for distinguishing GS ≤ 7 from GS ≥ 8 PC lesions was 0.710 (95% Cl 0.539; 0.881; SD (AUC) = 0.087; *p* = 0.055) with a SUV_max_ of 6.2 (subgroup: ^68^Ga-7/8) and with a sensitivity of 89%, a specificity of 33%, a PPV of 43%, and an accuracy of 63% (Table 3).

**Figure 3:**
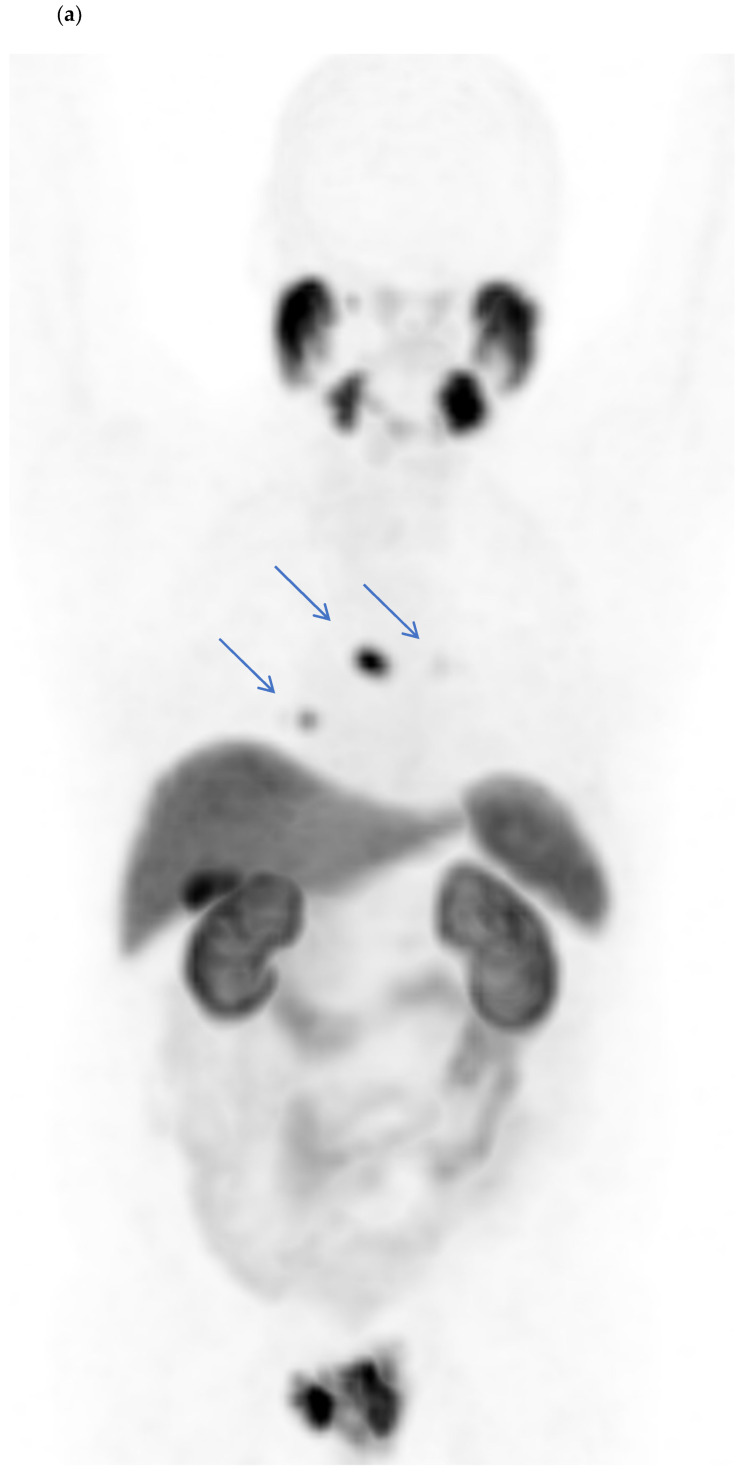

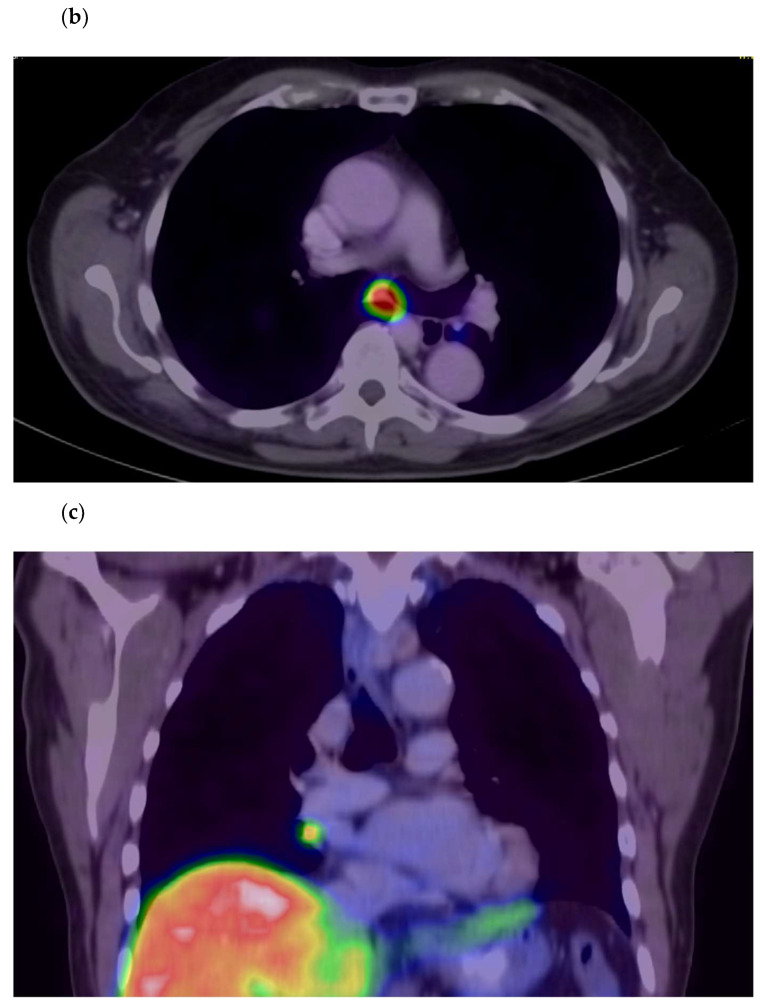
(**a**–**c**) Case study of a patient with evidence of a prostate-specific membrane antigen (PSMA)-avid prostatic finding in the initial staging (**a**) (with an initial prostate-specific antigen of 5.0 ng/mL) concordant with the histopathologically confirmed aggressive prostate carcinoma (PC) with a Gleason Score of 8 (4 + 4). The [^18^F]PSMA-1007 positron emission tomography/computed tomography showed no locoregional lymph node metastases or skeletal metastases, but three mediastinal lymph nodes ((**a**): blue arrows) of normal size, located infracarinally (**b**) and bilaterally hilar ((**c**): hilar right), carrying intensive tracer uptake (the highest maximum standardized uptake value of 11.4), which were histopathologically confirmed as metastatic PC.

**Figure 4:**
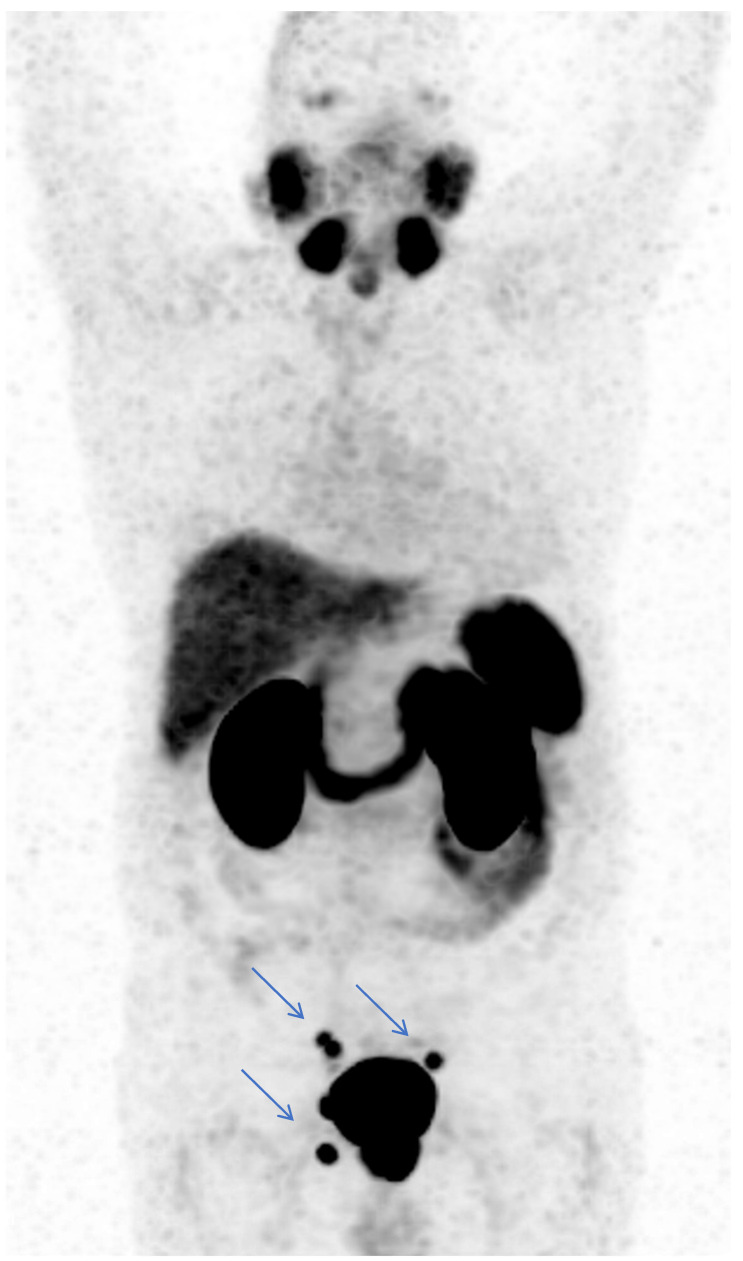
Case study of a patient with evidence of a prostate-specific membrane antigen (PSMA)-avid prostatic finding in the initial staging with an initial prostate-specific antigen of 13.0 ng/mL, concordant with the histopathologically confirmed aggressive prostate carcinoma (PC) with a Gleason Score of 8 (4 + 4). The [^68^Ga]Ga-PSMA-11 positron emission tomography/computed tomography showed five locoregional lymph node metastases (blue arrows) carrying intensive tracer uptake (the highest maximum standardized uptake value of 19.4), which were histopathologically confirmed as metastatic PC.

Figure 3 shows a ^18^F-PSMA with a histopathologically confirmed aggressive PC with a GS of 8 (4 + 4), without locoregional LNM and without skeletal metastases, but with three mediastinal LNM of normal size, located infracarinally and bilaterally hilar with a high PSMA avidity, and Figure 4 shows a ^68^Ga-PSMA with a histopathologically confirmed aggressive PC with a GS of 8 (4 + 4) with locoregional LNM and without distant LNM and without skeletal metastases.

## 4. Discussion

The EAU-EANM-ESTRO-ESUR-ISUP–SIOG Guidelines 2022 explicitly emphasize that most published studies on the primary staging of PC were based on ^68^Ga-labeling for PSMA PET imaging, and few studies were based on ^18^F labeling [2,7]. According to these guidelines, there are currently no conclusive data comparing ^68^Ga-PSMA with ^18^F-PSMA imaging in primary PC staging. In this context, the present study can possibly make a valuable contribution to the comparison of the two methods, ^68^Ga-PSMA and ^18^F-PSMA, in the clinical staging of PC.

In this comparative study of ^68^Ga-PSMA vs. ^18^F-PSMA in patients with newly diagnosed PC, we analyzed the PSMA-positive lesions that were determined to be malignant. PSMA-avid prostatic foci in concordance with histopathologically proven PC were found in all 52 study patients in the ^18^F-PSMA cohort, while ^68^Ga-PSMA showed them in 97.2% of the cohort (35/36). The imaging data for prostatic lesions were compared with histopathologic prostate biopsy results expressed as GS. Our results showed concordant findings with both tracers, which is in line with other studies comparing ^18^F-PSMA and ^68^Ga-PSMA in primary staging [8,9,10]. Kuten et al. reported in a head-to-head comparison that the identification of all intermediate- and high-risk PC lesions was comparable by both methods [8]. Hoberück et al. described, in a retrospective intraindividual comparison, that ^18^F- as well as ^68^Ga-PSMA appeared largely interchangeable, with neither tracer significantly outperforming the other [9]. The authors described that no significant difference considering SUV_max_ of tumor lesions was shown [9]. A prospective intraindividual comparative study on ^18^F-PSMA and ^68^Ga-PSMA for PC staging, evaluation at biochemical recurrence and assessment of metastatic disease, by Pattison et al. demonstrated a high concordance of 92% for TNM stage [10]. Further studies confirmed similar findings in PSMA PET/CT imaging with the two radiopharmaceuticals in the setting of restaging PC patients, too [11,12]. Rauscher et al. showed similar detection rates in patients with biochemical recurrence after radical prostatectomy. However, five times as many positive findings of benign origin were found in ^18^F-PSMA compared with ^68^Ga-PSMA [11]. The side-by-side evaluation specifically requested by the authors for the ^18^F-PSMA diagnosis of PET and CT images as well as intensive reader training on well-known pitfalls (for example, non-specific tracer uptake in the ganglia) in the clinical context [11] was implemented in a quality-assured manner by the diagnostic specialists in our present study. In a further restaging study by Hoffmann et al., both methods (^18^F-PSMA and ^68^Ga-PSMA) showed comparable overall findings [12]. Exceptions to this, however, were a clearer distinction between positive and negative results in the ^18^F-PSMA imaging considering a PSA threshold, determined in the study, in biochemical recurrent patients after radical prostatectomy [12]. However, Rahbar et al. described on the basis of patient images that ^18^F-PSMA offers an advantage over imaging with ^68^Ga-PSMA for the detection of local recurrence after primary local therapy due to the later renal tracer excretion. The authors related this advantage to case constellations with unclear lesions near the ureter or the urinary bladder [13]. Renal excretion of ^68^Ga-PSMA and radioactive bladder filling obscures local recurrence in the situation of biochemical recurrence but is of less relevance in initial tumor staging as in our study. Considering the comparison of ^68^Ga-PSMA and the PET/CT with another ^18^F-labeled radiotracer, named [^18^F]rhPSMA-7 (^18^F-rhPSMA-7), a study by Kroenke et al. showed similar tumor positivity rates and SUV_max_ values for primary PC and biochemical recurrence of PC [7,14]. Giesel conducted a comparative study considering different ^18^F-labeled PSMA PET ligands. The comparison of [^18^F]DCFPyl PET/CT (^18^F-DCFPyl) with ^18^F-PSMA also showed no significant differences in the detection of carcinoma foci or their SUV_max_ values [6].

In order to improve underdetection of high-grade PC and overdetection of low-grade PC [2,4], it makes sense to define a separation sharpness for the clinical setting. The cancer patients who would not benefit from a therapy should be considered separately from the patients with expected therapy success. The EAU-EANM-ESTRO-ESUR-ISUP–SIOG Guidelines 2022 do not specify how the term csPC should be defined exactly [2]. The guidelines report that studies mostly define GS 7 tumors and upwards or GS 7b tumors and upwards as clinically significant and that authors should decide for themselves and explain this in the study design [2]. In our study, we defined in one patient subgroup csPCa as any ISUP grade group ≥ 3 malignancy (patients with the high–intermediate or intermediate-unfavorable PC risk of GS 7b and above) and in a second patient subgroup csPCb as any ISUP grade group ≥ 4 malignancy (patients with the high PC risk of GS 8 and above), in order to then be able to compare both groups. Our study mainly focused on analyzing the best SUV_max_ cut-off value to identify the clinically significant PC foci and to compare the results of both methods. PSMA-avid lesions were defined as suspicious of malignancy when the uptake of the tracer was significantly higher than the surrounding benign tissue, when the tracer uptake appeared focal in character, and when the lesions were classified as primarily malignant (in the opinion of experts based on their extensive experience in the interpretation of PSMA PET/CT scans). Experience has shown that suspicious PET lesions with a SUV_max_ of 2.5 or higher were mostly associated with compatible and duplicatable visual evidence of PC foci and, therefore, this value was initially used as a cut-off to distinguish between PET positivity and negativity for both radiopharmaceuticals. Because the tumor-to-background ratio for the malignant lesions compared with the benign tissue in the PSMA PET/CT is very high according to previous studies (e.g., in comparison to FDG PET/CT, [15]) and the difference in the detected lesions was clearly shown in the present study, we did not list the SUV_mean_ values separately, as this would have no added value.

First, choosing a routinely used SUV_max_ of 2.5 as the cut-off value between csPC and clinically insignificant PC, the findings of both methods demonstrated similar concordance in our study. ^18^F-PSMA revealed 25% (13/52) of PC prostatic lesions with histopathology of low- and intermediate-favorable risk PC (GS ≤ 7a) vs. 75% (39/52) with histopathology of intermediate-unfavorable and high-risk PC (GS ≥ 7b) with a sensitivity of 100%, a PPV of 76%, and an accuracy of 76% considering a SUV_max_ of 2.5. For ^68^Ga-PSMA, the results were 31.4% (11/35) vs. 68.6% (24/35) with a sensitivity of 97%, a PPV of 75%, and an accuracy of 77% with the uptake of the radiotracer above a SUV_max_ of 2.5. In the present study, because the specificity of both methods was extremely low (10% vs. 27%) using a SUV_max_ threshold of 2.5, an optimal SUV_max_ cut-off value was determined for ^18^F-PSMA and for ^68^Ga-PSMA by Youden index calculation. The reasons for reduced specificity in PSMA imaging are well known and include neovascularization and PSMA overexpression in non-prostatic tissue, e.g., benign neoplasms, i.e., thyroid and parathyroid adenomas, and in non-prostatic malignancies such as breast cancer, thyroid cancer, gliomas, lung cancer, neuroendocrine tumors, lymphoma, and renal cell carcinoma. There are fewer false positives if the PSMA images are interpreted by experts who are aware of the various pitfalls [16].

Subsequently, ROC curves were used to characterize the diagnostic performance. By considering the PSMA-avid prostatic lesions and the corresponding classification in the GS based on the biopsy, a SUV_max_ of 8.95 was analyzed by ROC analysis (*p* = 0.007) to differentiate between csPC and clinically insignificant PC (subgroup: csPCa) for ^18^F-PMSA with a sensitivity of 62%, a specificity of 85%, a PPV of 92%, and an accuracy of 67%. ^68^Ga-PSMA gave similar findings for a SUV_max_ of 8.7 (*p* = 0.003) with a sensitivity of 54%, a specificity of 91%, a PPV of 93%, and an accuracy of 66%, respectively. However, our data show a higher (but also moderate) specificity and a higher PPV for ^18^F-PSMA (52% and 61% based on a SUV_max_ of 4.75) in comparison with ^68^Ga-PSMA (33% and 43% based on a SUV_max_ of 6.2), when differentiating between low- and intermediate-risk PC vs. high-risk PC (subgroup: csPCb), with comparable sensitivity (90% vs. 89%) and accuracy (63% both). But these data did not show statistical significance (SUV_max_ of 4.75, *p* = 0.26 and SUV_max_ of 6.2, *p* = 0.055). Kuten et al. calculated ROC curves to distinguish pathological from non-pathological components of the prostate, for which both methods proved to be suitable [8]. A comparison of the results with our calculated values is not possible because the comparison groups differ. Additionally, due to the lack of statistical significance, no optimal SUV_max_ values could be calculated in the study by Kuten et al. [8].

The results of diagnostic PSMA imaging as part of the staging of PC offer the possibility of guiding biopsy and therapy management to detect the targeted PC lesions with the most aggressive tumor foci (csPC) [17,18]. A mpMRI in combination with a PSMA hybrid imaging fusion biopsy could increase the accuracy of directed biopsy [18]. Pepe et al. demonstrated a lower false positive rate and a better negative predictive value compared with mpMRI. In 80% of the cases, a biopsy could have been omitted based on the PSMA PET/CT results [18]. As part of individual therapy management, hybrid imaging with PSMA PET/CT enables optimal patient selection as well as personalized monitoring [17]. In this regard, our calculated SUV_max_ cut-offs can be used to differentiate between low- and intermediate-favorable from intermediate-unfavorable and high-risk PC lesions. The more we know about diagnostic imaging (such as the correlation between PSMA receptor density and GS as well as PSMA imaging with different radiopharmaceuticals and their physiological expression in non-prostatic benign tissue and non-prostatic tumors, both benign and malignant) and can optimize it, the better therapy decisions can be made [17]. Because present EAU Guidelines state that there is currently no conclusive data comparing ^68^Ga-PSMA vs. ^18^F-PSMA imaging in primary PC staging [2], we investigated this. The comparison could not show any clear advantage for one of the methods in our study, which is also an important statement for clinical application. In all 52 study patients in the ^18^F-PSMA cohort and in 97.2% (35/36) of the patients in the ^68^Ga-PSMA cohort, PSMA-avid prostate lesions were detected concordant with histopathologically proven PC. PSMA-positive metastases were shown in 5.8% (3/52) in the intermediate-favorable risk ^18^F-PSMA cohort vs. in 30.8% (16/52) in the intermediate-unfavorable risk group, but no PSMA-avid metastases (0/35) were seen in the ^68^Ga-PSMA intermediate-favorable risk cohort vs. 25.7% (9/35) with GS ≥ 7b. In view of the nearly similar results and the good performance of ^18^F- as well as ^68^Ga-labeled compounds, the challenge for the use of the appropriate radiopharmaceutical could potentially be made depending on availability [12]. Nevertheless, further studies are needed to assess the position of routinely established ^68^Ga- and ^18^F-labeled compounds in PSMA imaging and their actual clinical utility. These will be carried out on the different radiotracers in order to shed light on new aspects, the overall impact on survival, and the clinical impact of PSMA-based diagnostics such as PSMA-targeted biopsies [7,18]. In this context, a randomized study that would perform a combined PSMA imaging with a mpMRI as a guide for prostate biopsy in the initial stage with a high suspicion of csPC and would consider different radiotracers might be useful [19,20,21,22]. Limitations of the present study include the retrospective nature of the analysis, the small number of patients, and the lack of an intraindividual comparison of the patients. To confirm and expand our results we recommend further studies, ideally prospective with larger patient cohorts.

## 5. Conclusions

^18^F-PSMA and ^68^Ga-PSMA both show promising results in the detection of newly diagnosed PC with comparable correlation of PSMA-avid lesions with GS. Neither method showed an outstanding superior advantage. Studies reporting ^18^F-PSMA and ^68^Ga-PSMA are equally relevant for the staging of patients with PC. With regard to both methods, the importance of PSMA imaging for the detection of metastases is also clear in primary staging, especially in patients with high-risk and intermediate-unfavorable risk PC. Our calculated thresholds for the SUV_max_ value may represent valuable parameters in clinical use for the discrimination of csPC from non-csPC and may also serve to guide prostate biopsies and support the identification of aggressive PC foci. 

## Figures and Tables

**Table 1:** Patients’ characteristics.

Clinical Variable	Value	Clinical Variable	Value
Number of [^18^F]PSMA-1007 PET/CT patients	52	Number of [^68^Ga]Ga-PSMA-11 PET/CT patients	36
Age		Age	
Median	67.5	Median	65.5
Range	51–80	Range	48–79
Mean ± SD	67.4 ± 7.7	Mean ± SD	65.8 ± 7.7
Gleason Score (GS)		Gleason Score (GS)	
GS 6	3	GS 6	5
(low-risk + grade group 1)	5.8%	(low-risk + grade group 1)	13.9%
GS 7a	10	GS 7a	7
(low–intermediate or intermediate-favorable risk + grade group 2)	19.2%	(low–intermediate or intermediate-favorable risk + grade group 2)	19.4%
GS 7b	11	GS 7b	14
(high–intermediate or intermediate-unfavorable risk + grade group 3)	21.2%	(high–intermediate or intermediate-unfavorable risk + grade group 3)	38.9%
GS 8	8	GS 8	7
(high-risk + grade group 4)	15.4%	(high-risk + grade group 4)	19.4%
GS > 8	20	GS > 8	3
(high-risk + grade group 5)	38.5%	(high-risk + grade group 5)	8.3%
PSA (ng/mL)		PSA (ng/mL)	
Median	8.8	Median	13.0
Range	2.68–167	Range	3.1–93
Positivity rate		Positivity rate	
PET/CT positive	52/52	PET/CT positive	35/36
patients/total	100%	patients/total	97.2%

Abbreviations: PSMA, prostate-specific membrane antigen; PET/CT, positron emission tomography/computed tomography; SD, standard deviation; y, year; PSA, prostate-specific antigen.

**Table 2:** PSMA-positive scan lesions for staging patients in relation to the Gleason Score (GS).

	GS < 7	GS 7a	GS 7b	GS 8	GS > 8	Chi^2^, *r*
[^18^F]PSMA-1007 PET/CT patients (52):						
PSMA positive (52/52)	3	10	11	8	20	
Prostatic lesions (52/52)	3/5.8%	10/19.2%	11/21.2%	8/15.4%	20/38.5%	
Metastases (19/52)	0	3/5.8%	3 /5.8%	3/5.8%	10/19.2%	*p* =0.494 * r = 0.252
LNM (17/52)	0	2/3.8%	3/5.8%	3/5.8%	9/17.3%	*p* = 0.531 * r = 0.266
[^68^Ga]Ga-PSMA-11 PET/CT patients (36):						
PSMA positive (35/36)	4	7	14	7	3	
Prostatic lesions (35/36)	4/11.4%	7/20%	14/40%	7/20%	3/8.6%	
Metastases (9/36)	0	0	3/8.6%	4/11.4%	2/5.7%	*p* =0.030 * r = 0.513
LNM (6/36)	0	0	2/5.7%	2/5.7%	2/5.7%	*p* = 0.086 * r = 0.442

* Fisher exact test. Abbreviations: PSMA, prostate-specific membrane antigen; PET/CT, positron emission tomography/computed tomography; LNM, Lymph node metastases; GS, Gleason Score; *p* < 0.05 is considered significant; *r*, Pearson correlation coefficient.

**Table 3:** Test parameters for the staging of prostate cancer with [^18^F]PSMA-1007 PET/CT and with [^68^Ga]Ga-PSMA-11 PET/CT; distribution of positive prostatic findings classified by Gleason Score (GS) categories (GS ≤ 7a versus GS ≥ 7b; GS ≤ 7 versus GS ≥ 8).

	GS ≤ 7a vs. ≥7b Cut-Off SUV_max_ 2.5		GS ≤ 7a vs. ≥7b Cut-Off SUV_max_ 8.95/SUV_max_ 8.7 *		GS ≤ 7 vs. ≥8 Cut-Off SUV_max_ 4.75/SUV_max_ 6.2 **	
	[^18^F]PSMA-1007 PET/CT	[^68^Ga]Ga- PSMA-11 PET/CT	[^18^F]PSMA-1007 PET/CT	[^68^Ga]Ga- PSMA-11 PET/CT	[^18^F]PSMA-1007 PET/CT	[^68^Ga]Ga- PSMA-11 PET/CT
Sensitivity	100%	97%	62%	54%	90%	89%
Specificity	10%	27%	85%	91%	52%	33%
NPV	100%	100%	42%	48%	73%	93%
PPV	76%	75%	92%	93%	61%	43%
Accuracy	76%	77%	67%	66%	63%	63%

Abbreviations: SUV_max_, maximum standardized uptake value; vs., versus; PSMA, prostate-specific membrane antigen; PET/CT, positron emission tomography/computed tomography; NPV, negative predictive value; PPV, positive predictive value. * for [^18^F]PSMA-1007 PET/CT SUV_max_ 8.95, [^68^Ga]Ga-PSMA-11 PET/CT SUV_max_ 8.7. ** for [^18^F]PSMA-1007 PET/CT SUV_max_ 4.75, [^68^Ga]Ga-PSMA-11 PET/CT SUV_max_ 6.2.

## Data Availability

The datasets analyzed during the current study are available from the Practice of Radiology and Nuclear Medicine in Cologne/Germany, named “Praxis im KölnTriangle” upon reasonable request.
